# Current Status and Future Prospective for Nitrogen Use Efficiency in Wheat (*Triticum aestivum* L.)

**DOI:** 10.3390/plants11020217

**Published:** 2022-01-14

**Authors:** Anamaria Mălinaş, Roxana Vidican, Ioan Rotar, Cristian Mălinaş, Cristina Maria Moldovan, Marian Proorocu

**Affiliations:** 1 Department of Plant Culture, Faculty of Agriculture, University of Agricultural Sciences and Veterinary Medicine, 400372 Cluj-Napoca, Romania; anamaria.malinas@usamvcluj.ro (A.M.); roxana.vidican@usamvcluj.ro (R.V.); ioan.rotar@usamvcluj.ro (I.R.); 2 Department of Environmental Protection, Faculty of Agriculture, University of Agricultural Sciences and Veterinary Medicine, 400372 Cluj-Napoca, Romania; marian.proorocu@usamvcluj.ro

**Keywords:** nitrogen use efficiency, nitrogen uptake, wheat, sustainable development, fertilizer N

## Abstract

Although essential for achieving high crop yields required for the growing population worldwide, nitrogen, (N) in large amounts, along with its inefficient use, results in environmental pollution and increased greenhouse gas (GHG) emissions. Therefore, improved nitrogen use efficiency (NUE) has a significant role to play in the development of more sustainable crop production systems. Considering that wheat is one of the major crops cultivated in the world and contributes in high amounts to the large N footprint, designing sustainable wheat crop patterns, briefly defined by us in this review as the 3 Qs (high quantity, good quality and the quintessence of natural environment health) is urgently required. There are numerous indices used to benchmark N management for a specific crop, including wheat, but the misunderstanding of their specific functions could result in an under/overestimation of crop NUE. Thus, a better understanding of N dynamics in relation to wheat N cycling can enhance a higher efficiency of N use. In this sense, the aim of our review is to provide a critical analysis on the current knowledge with respect to wheat NUE. Further, considering the key traits involved in N uptake, assimilation, distribution and utilization efficiency, as well as genetics (G), environment (E) and management (M) interactions, we suggest a series of future perspectives that can enhance a better efficiency of N in wheat.

## 1. Introduction

The use of synthetic fertilizers in agriculture is fundamental for sustaining the growing population worldwide. Recent reports show that the amount of mineral N fertilizer applied in agriculture in the European Union (EU; all the abbreviations used in this manuscript can be found in Table S1) increased from 10.8 million tons in the year 2011 to 11.6 million tons in the year 2017, while European cereal production increased only from 270.8 thousand tons to 284.9 thousand tons [1]. The excessive and inefficient use of fertilizer N is a global issue that requires not only high production costs, but results in environmental pollution and the increase in GHG emissions. Currently, the challenge is to maintain or improve productivity and profits with reduced inputs, essentially, to farm more cleanly and efficiently [2]. The current reports on climate change issues highlighted the important contribution of agriculture to increasing the emissions of GHGs. In fact, agriculture contributed to about 20% of the total global anthropogenic emissions in 2017, roughly 75% of all nitrous oxide (N_2_O) emissions and 42% of all methane (CH_4_) emissions in 2017 [3]. According to the latest inventory data published by the European Environment Agency (EEA) for EU agriculture, the land use and land use change (LULUC) of grassland and cropland made up 12% of all GHG emissions in 2016 [4]. As a consequence, most of the protocols related to climate change mitigation demand sustainable agricultural systems. In order to achieve the ambitious objective to transform the EU’s space in the first zero emissions continent by the year 2050, we need to make more efforts to achieve the synchrony between the N supply and crop demand without excess or deficiency [5,6]. The most recent Common Agricultural Policy (CAP), formulated in June 2021, which should be implemented starting from 2023, shows a strong emphasis on results and performance, and aims to foster a sustainable and competitive agricultural sector that can support the livelihoods of farmers and provide healthy and sustainable food for society [7]. Feeding a fast-growing world population still remains a challenge with the current production patterns [8].

Cereals are the most important sources of calories and nutrition for the human population and are an essential source of animal feed [9]. Wheat is the most important cereal crop in the world, totaling an average harvested area of almost 215 million hectares in the year 2018 [10], owing to its adaptability to a wide range of environments [11,12]. Nitrogen fertilizer is a costly component during wheat crop production, and excess N can lead to negative environmental repercussions [13,14]. According to the Food and Agriculture Organization (FAO) [15], 38.1 MMT of fertilizer N was globally applied to all crops, while one-third of this amount was used just for the wheat crop. Research conducted thus far, highlights that the intensive agricultural production systems, which are still applied in a high proportion all around the world, are subjected to N loss. Moreover, it is estimated that between 50% and 75% of the N applied to the field is not used by the plant and is lost by leaching into the soil [16,17]. Therefore, the current recommendations are to move away from the recipe-type approach of applying flat rates of fertilizer N throughout the year, to a more informed approach allowing for a better outcome with respect to wheat production, NUE and N losses [18].

For a large number of crops, including wheat, there is a genetic variability for both N absorption efficiency and for NUE [17]. Field studies have proven that the best performing crop varieties at a high N fertilization input are not necessarily the best ones when the supply of N is low [17]. It is estimated that globally only 33% of applied fertilizer N is recovered in the harvested grain, which is indicative of a large waste of resources and a potential major source of pollution, and is thus a major target for crop improvement [9]. Thus the dynamics of N availability in the wheat plant (within the plants, canopy and root), should be considered for a better understanding of the capacity of crops to capture, allocate and use N for optimizing yields.

Moll et al. [19] highlighted that one way to improve yield limited by N without disrupting the environment is to enhance crop NUE. According to Cassman et al. [5], NUE of a cropping system can be defined as the proportion of all N inputs that are removed from harvested crop biomasses, contained in recycled crop residues and incorporated into soil organic matter and inorganic N pools. Various indices are commonly used in agronomic research to assess the efficiency of applied N [5,20], mainly for purposes that emphasize crop response to N. Nonetheless, there are numerous approaches to calculate the NUE and the current concerns are related to their shortcomings, which could result in a misunderstanding of their specific functions [21].

Our analysis explores the NUE in wheat, the most important cereal crop in the world, which also accounts for the majority of global N fertilizer use. With the final aim to identify the major pathways that can enhance a higher NUE and a sustainable pattern for the wheat cropping system, briefly defined by us through the 3 Qs (delivering high quantity yields, good quality products and the quintessence of natural environment health), we provide a thorough analysis of the current knowledge related to the capacity of wheat to capture, allocate and use N for optimizing yields and quality along with G × E × M interactions.

## 2. Nitrogen Use Efficiency Indices

NUE is a critically important concept for evaluating wheat crop production [22], whether the N source is a commercial fertilizer, manure or other forms, and indicates the potential for nutrient loss in the environment from cropping systems.

The efficiency of applied N can be assessed through various indices, categorized by Congreves et al. [21] into groups, such as fertilizer-based, plant-based, soil-based, ecology-based or system-based NUEs. To date, several reviews on NUE assessments and calculations are available in the scientific literature [21,22,23]. One of the most important aspects when using NUE indices, is that none of these available indices are without their faults, as we will detail further. Therefore, it is recommended that one should use more than one index to evaluate the NUE of a specific crop, including wheat. While some indices take into account the majority of N sources, some NUE indices do not consider some important N pools, such as the background soil N levels or indices that fail to provide clear and detailed information about the performance of the cropping system or N cycling over time. Thus, although using these indices might appear simple at first sight, the selection of the most appropriate indices should rely on the main hypotheses of our research. Therefore, when selecting the most appropriate NUE indices, one should consider the main questions addressed by its research along with crop specificities. Table 1 comprises the major NUE indices and their respective definitions, after Congreves et al. [21], Fixen et al. [22] and Dobermann [23].

The major purpose for NUE index assessment is/should be to find out how N management can be shaped to improve the overall crop productivity, by providing the optimum nutrient supply to the crop while minimizing the nutrient losses from the field. NUE is primarily composed of N uptake efficiency and N utilization efficiency [19], and their assessments require the measurements of grain yield (GY), grain N concentration (GPC), straw yield, aboveground, non-grain biomass and the straw N concentration [24]. The components of the grain protein yield are GY and GPC, and these two traits are the two principle targets of most hard wheat improvement programs [25,26].

The most commonly used NUE indices are the fertilizer-based and plant-based NUEs (Table 1). In general, most of these indices are suited to short-term time scales, such as growing seasons. While their assessments can provide valuable crop- and site-specific information, these indices provide limited information regarding the inferences that can be made across a crop rotation, or about the performance of the cropping system or N cycling over time [21].

The fertilizer-based NUE indices are defined by the amount of fertilizer applied in relation to various plant parameters, such as aboveground biomass, yield or N content [21,22]. One of the most significant limitations of these indices is that some of them do not consider the background soil N levels and, therefore, their use can provide a misunderstanding of NUEs if the soil N reserves in the non-fertilized control plots are depleted over long-term periods [21]. The fertilizer-based NUE indices include several indicators, such as AE, PFP, PNB and RE (Table 1). The AE quantifies the total economic output in relation to the utilization of all nutrient resources in the system. Frequently, the highest AE is recorded at the lowest fertilizer rates [21]. The typical AE values for wheat, as reported by Fixen et al. [22], range between 15 and 30 kg of grain kg nutrient^−1^. Nonetheless, AE can be useful when focusing on plants yield, and the use of this indicator in short-term trials can underestimate the AE by neglecting the residual effects of repeated fertilizer applications [21]. PFP is an NUE index showing the productivity levels of the cropping system in relation to the fertilizer N input. The typical PFP values for wheat range between 40 and 90 kg of grain kg nutrient^−1^ [22]. The shortcomings of PFP are mainly expressed by the fact that this indicator does not account for background (indigenous) soil N, and thus cross-site comparisons are limited by neglecting to account for background soil N [21]. PNB is the NUE index expressed as the nutrient output (taken out of the system) in relation to the nutrient input (a ratio of “removal to use”). The typical PNB values for wheat, as reported by Fixen et al. [22], range between 0.7 and 0.9 kg of grain kg nutrient^−1^. Similar to PFP, PNB limitations are induced by the fact that its calculation does not account for background soil N. RE is the NUE index expressed as the difference in nutrient uptake in aboveground parts of the plant between the fertilized and unfertilized crop in relation to the quantity of nutrients applied. The typical RE values for wheat, as reported by Fixen [22], range between 40 and 65%. The shortcomings of this index are the result of its inability to be used in trails without a non-fertilized plot.

The plant-based indices are defined by plant tissue N in relation to crop yield or yield N. Their calculation is useful, especially for the breeding programs focusing on identifying plant genotypes with the enhanced capability of allocating growth or N resources towards the economic portion of plants [22]. This group includes PE, NUtE, IE and NHI (Table 1). PE is defined as the fertilizer N from the plant tissues in relation to the yield recorded. The major limitations expressed by this index rely on its inability to be applied to long-term trials, as the depletion of indigenous soil N in non-fertilized controls can lead to erroneously high NUEs [21,23]. NUtE is defined as the N content in the yield, in relation to the N content found in plant tissues. IE is the NUE index expressed as the yield in relation to the total nutrient uptake. The typical IE values for wheat, as reported by Dobermann [23], range between 30 and 66 kg grain of kg nutrient^−1^. The limitations of this index are highlighted by the fact that it does not account for background soil N, and thus the results can be misinterpreted because the high IE values may indicate the N deficiency rather than the increased NUE [23]. The NHI has the same signification and limitations as the IE; the only difference being that this index is expressed in percentages.

The soil-based indices are defined by the fertilizer N inputs and soil N contributions to the plant components as being primarily influenced by the soil management and N dynamics [21]. This group includes a wide range of indices. Such as NUpE and NUE_yield_. NUpE is defined as the percentage of available soil N that is utilized by the plant. This index weak point is that it does not account for the soil N mineralized throughout the growing season [21].

The ecology-based indices describe the dry weight that can be produced per unit of N taken up, under steady-state conditions [21]. This group includes NP and NUEecology (Table 1). The NP provides a meaningful understanding of the plant’s immediate NUE, but its weak point is that it is not helpful in providing an insight into the plant’s long-term or potential performance in agroecosystems.

The system-based indices are defined by the spatial and temporal boundaries, formulated as a balance or a difference [21]. This group includes several indices, among which sNBI and sNUE are the most useful in assessing wheat crop NUE (Table 1).

## 3. Key Traits for Wheat NUE

NUE is a critically important concept in the evaluation of wheat production and is highly influenced by fertilizer management, as well as by soil- and plant-water management [22]. This index is regulated by biological, physiological, environmental, genetic, agronomic and developmental factors [17]. Thus, a thorough understanding of N cycling in the soil–plant–atmosphere components of wheat cropping systems is an essential prerequisite for developing material with reduced N requirements [27,28], and there is a need for assessment in the context of the full plant and crop cycle along with the associated agronomy [27].

The research conducted thus far highlights that not all of the soil and fertilizer N can be accounted for at the end of a growing season [28]. In fact, it is already clear that the N concentration decreases with time during the growing season [29,30] because of the slower rate of N assimilation relative to C. Thus, relatively large losses of N from wheat during the early grain-filling period occurs immediately after anthesis has been reported [30,31]. The peak for wheat N demand occurs during the stem elongation stage; therefore, a deficiency in N during twining and flowering stages will negatively impact the yield. This type of response can influence N balance studies and crop NUE [28].

**Figure 1:**
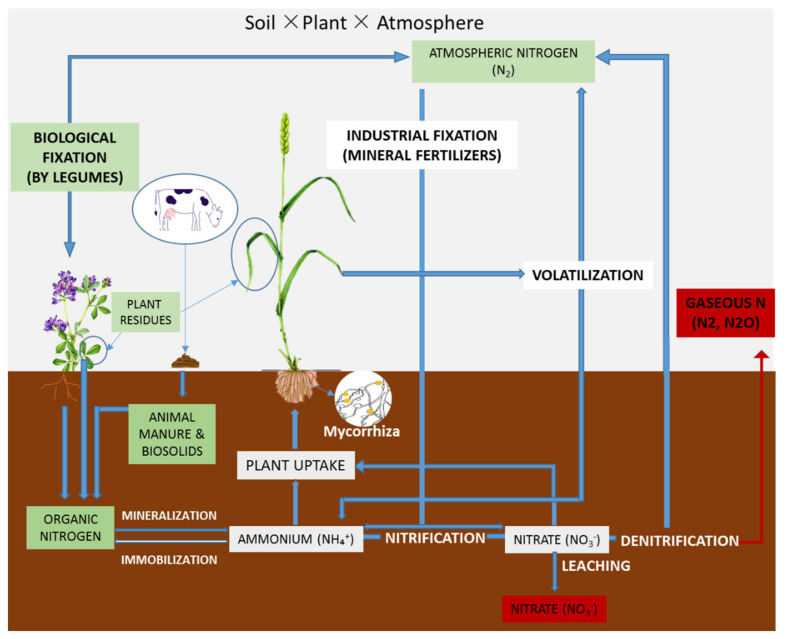
Soil × plant × atmosphere interactions during the N cycle in the wheat crop (original).

The two forms of N inputs in wheat agrosystems are organic and synthetic nitrogen (as described in Figure 1). The organic nitrogen originates from atmospheric nitrogen (N_2_), made available to plants through biological fixation by legumes, animal manure and biosolids, and crop residues [32,33]. Nitrogen originated from crop residues can vary between cultivars in terms of availability, being highly influenced by root traits and the interaction between roots and the soil [34]. The decomposition of plant and animal residues in soil results in some of the C and N being mineralized. The net mineralization of C normally begins immediately after soil supply with organic matter, and results in some CO_2_ release into the atmosphere, while most of the mineralized N remains in the soil–plant system, as a result of both ammonium and nitrate being readily soluble in water and their susceptibility to the uptake by plants and microorganisms [35]. Soil quality and fertility is related to the functionality and metabolism of soil microorganisms, as they influence and, at the same time, are influenced by the soil C and N contents, bacteria being an essential part of C and, even more so, of N cycling processes [36,37,38,39,40].

### 3.1. N Uptake, Assimilation and Use Effiency in Wheat

A key trait for wheat NUE is represented by the ability of the crop to take up nitrogen [9,41,42]. As shown in Figure 1, N is absorbed from the soil through the plant roots. The efficiency of the N uptake has been reported to be a genotype characteristic [43] and is attributed to the morphological, physiological and biochemical processes occurring in plants, as well as to their interaction with soil–climatic factors, fertilizer, biological and management practices [44,45]. Thus, N uptake is affected by the N supply in such a manner that, at low N rates, N uptake will rely on the morphological and physiological root characteristics while, at high N rates, N uptake will rely on the growth-related demand for N.

The N uptake is a function of the root structure, architecture and function [9,46,47], and implies nutrient movement from the surrounding soil to the root surfaces by diffusion or mass flow. The efficiency of this acquisition is highly influenced by root hairs [48]. Recently, Takahashi and Muhuddin [49], cited by Marschner and Rengel [50], revealed that nitrate moves by diffusion, approximately 3 mm in a day, potassium, about 1 mm in a day and phosphate, only about 0.1 mm in a day.

It was found that more developed and deeper roots can enhance an increase in the N uptake from deeper soil layers and reduce nitrate leaching losses in the environment [51,52]. Studies of the relationship between the root growth and N availability have shown that root proliferation is dependent on canopy formation and nitrogen status [53,54]. Robinson [55] highlighted that wheat roots tend to proliferate in nutrient-rich zones and are suppressed in zones with a low nutrient supply. The same research concluded that this response of the roots in relation to nitrogen status, enables plants to partially compensate for non-uniform supplies of nutrients [55]. Moreover, [56,57] this behavior may give single plants a competitive advantage against their neighbors, but it will have a limited effect on the overall crop N capture.

Considering root architecture, the studies conducted to date reported that a deeper root growth is more important in relation to the N capture than an increased root density [58,59,60]. This is mainly explained through the fact that deeper roots allow the crop to access N resources from deeper soil layers. According to Rasmussen et al. [61], the most important architectural influence is height that is influenced by dwarfing genes.

The field studies conducted to date, reported root growth in up to 1.5–2.0 m of soil depth for winter wheat [62,63,64,65,66]. According to Gao et al. [67], increased root growth in deep soil layers can be a response to higher subsoil N availability due to nitrate leaching. Controversially, several researches conducted to date revealed that winter wheat root densities increase when N fertilizer is applied [68]. More specifically, Rasmussen et al. [61] concluded in a field study that the winter wheat root density was increased by N fertilization by up to 150 kg N ha^−1^; above this level the effect varied between the two years analyzed. The same authors underlined that the most direct effects of N supply on root density were observed in the soil layers below 0.5 m, and that N fertilization did not appear to affect the root depth. Therefore, more studies should address this issue in order to reveal the optimal N fertilizer rate that could increase nitrogen uptake efficiency.

N is absorbed from the soil, mainly in the form of nitrate and ammonium. The efficiency of these two assimilation of the N forms is influenced by the plant species, age, environment, carbon hydrates supply, the presence or absence of some cations and anions in the soil solutions and the soil pH. In the soil, nitrate moves mainly by mass flow with the movement of water and partly by diffusion, whereas ammonium moves mainly by diffusion and only slightly by mass flow [37]. The rate of movement of the solution by mass flow depends on the rate of transpiration of the plant, and by soil water content and soil texture [69].

Considering the importance of these two N forms during the wheat plant life cycle, it seems that the nitric forms are more efficient in the first vegetation period (germination), while the ammonium forms express their influence mainly on the oxidation or redox processes developed during the tissue formation phase and N storage pools and can be harmful in high amounts during germination. The rate of uptake of both ammonium and nitrate by the roots is influenced by the temperature and pH but is different for the two ions. The pH of the growth medium tends to become more alkaline when nitrate is taken up by roots, whereas in the case of ammonium the pH tends to become more acidic. Such changes in soil pH can influence the availability of other nutrients, such as phosphorus expression, and is therefore a secondary effect on plant growth [37].

N uptake is also influenced by the concentrations of these two forms of N in the soil solution or in the nutritive solution, and by the presence or absence of some anions/cations. For example, ${\mathrm{NH}}_{4}^{+}$ is more efficiently absorbed by plants in solutions with low concentrations, in the form of chloride and sulfates, and when Ca^2+^ is found in the nutritive environment, while an average amount is absorbed in the presence of Mg^2+^ and very low in the presence of K^+^ and Na^+^. Among the anions affecting ${\mathrm{NH}}_{4}^{+}$ absorption by the plant, Cl^−^ is the most influential one. At the same time, the chlorine can have a negative effect on ${\mathrm{NH}}_{4}^{+}$ assimilation because it delivers new salts, some of them being harmful to the plants, such as the aluminum oxichloride (in acid soils). Ultimately, the presence of Ca^2+^ in high amounts in the nutritive environment is required for ammoniacal nutrition, whilst it is less favorable to nitrate nutrition.

Nitrate absorption by the plant roots affects the soil pH, which tends to become more alkaline, with the nitrate ion in effect being exchanged for a hydroxyl ion. The nitrate absorption from the soil through the plant roots is partly translocated to the shoots as nitrate, and part of it is reduced to ammonium in the plant roots or leaves. The reduction process is catalyzed by specific enzymes (nitrate and nitrite reductase) and involves giving up eight electrons and a source of energy, which in leaves comes from natural light through the carbon–hydrate cycle that transfers these eight electrons from chloroplasts to cytoplasm, in the presence of nitrate reductase. The activity of the nitrate reductase enzymes in roots tends to diminish as the roots mature, and the proportion of absorbed nitrate that is transported unchanged therefore tends to increase with the increasing maturity of the root system [70]. The reduction in ammonium takes place in the chloroplast, under the influence of nitrite reductase and ferredoxin. If ${\mathrm{NO}}_{3}^{-}$ is metabolized in the leaves, this is accompanied by some cations, such as K^+^, Ca^2+^ and Mg^2+^, which neutralize an important number of organic acids resulting from the metabolism process. Once the plant species age, the amino acids and organic acids migrate from the old leaves to the younger ones, or to the other reservoir organs, accompanied preferentially by K^+^, and thus enhance the content of Ca^2+^ and Mg^2+^ in the old leaves. The nutrition with nitric N increases the amount of organic acids in the plants, while the supply with ammoniacal N results in the formation and accumulation of reduced compounds in plants. What does it mean? In practice, this means that the plants that are firstly supplied with nitric N will have a better ability to absorb the ammoniacal N later, and vice versa.

The assimilated nitrate is transported among the root cells by a set of plasma–membrane localized transporters, and those that are among the most important for the N uptake in wheat are the NITRATE TRANSPORTER 1/PEPTIDE TRANSPORTER family (NPF) and NITRATE TRANSPORTER 2 family (NRT2) [9,71]. It has been shown that, generally, NPFs have low nitrate affinities, while NRT2 transporters have a high affinity for nitrate, being induced under nitrate limiting conditions [72,73,74]. NPFs are also involved in the transportation of other divergent substrates, including peptides, hormones and glucosinolate [19,75]. Lupini et al. [76] evaluated the NUE in four ancient and four modern wheat genotypes and concluded that the ancient varieties exhibited higher PANU values compared to the modern ones; their behavior was also supported by the main N uptake-related gene expression analysis (NRT2.1, NPF6.3).

The second major N source ammonium is absorbed by the plant in the presence of an energy source (adenosine triphosphate/ATP) and enzymes, such as glutamate synthase and transaminases (which reduce, at the same time, both the ferredoxin and the piridinucleotides), and is further transformed in α-amino acids. Ammonium absorption by the plant roots affects the soil pH, which tends to become more acidic, with the ammonium ion in effect being exchanged for a hydrogen ion.

A good assimilation of ammoniacal N is directly influenced by the supply of carbon hydrates to the plant species. Following assimilation, the ammonium forms are transported into the root cells by a set of ammonium transporters in the plants (AMTs). To date, six AMTs have been identified in Arabidopsis, three of which (AtAMT1;1, AtAMT1;2 and AtAMT1;3) are involved in the uptake of approximately 90% of ammonium [77].

The ${\mathrm{NH}}_{4}^{+}$ accumulated in high amounts can be toxic for plants, and plant leaves can be affected when the long-term average concentration in the atmosphere is greater than about 75 μg NH_3_ m^−3^ [78]. In contrast, at low concentrations found normally in the atmosphere, plant leaves with a temporary surplus of N can release ammonia into the atmosphere.

The assimilated N is further allocated to the N uptake in five plant compartments: the leaf blades, stems and leaf sheaths, roots, grains and a reserve pool of primary photosynthetic products [79]. A close relationship has been noted between the N supply, leaf N distribution and leaf photosynthesis [77].

Nitrogen is first utilized to produce an effective canopy [9], which affects the grain filling and expresses a wide genetic variation within the wheat species [7,19]. According to Jenner et al. [80], leaf senescence is induced by N demands for grain filling. The metabolic changes involved in senescence are controlled genetically, but the timing of senescence and its rate of progress are influenced by the nutrient supply, light intensity and water stress. An important aspect concerning leaf senescence is the change of enzyme activity, thanks to the fact that the enzymes involved in assimilation lose their activity while those involved in degradation gain in activity. During the later stages of senescence, a disintegration of membrane tissue occurs, further affecting the translocation of N in phloem. Thus, delaying leaf senescence through the remobilization of stored N in preference to photosynthetic N from vegetative tissues is desirable, since it can enhance a better yield and higher grain protein [81].

NUE is influenced by the canopy height and flowering time as well, and according to Bai et al. [82], this can include the impacts on root proliferation mediated by *rht* genes, which further impacts the effectiveness of the roots in nitrogen acquisition.

Several field studies were conducted, to date, to track the N requirement for an optimal canopy of winter wheat. Sylvester-Bradley and Kindred [25] reported that, for 95% of light interception, the N requirement is 3 g N m^−2^ in a green area. Sinclair and Horie [83] highlighted that the maximum rates of photosynthesis in C also occur at leaf N concentrations above 2 g N m^−2^ in a green leaf.

Generally, a high N supply favors the excessive growth of vegetative plant parts enhancing the modification of the report between the aerial plant part and roots with a high influence on fructification processes. In the particular case of wheat crops, the studies developed to date revealed that in conditions of high N supplies, wheat plants tend to accumulate and store high amounts of N, regardless of whether or not these are required for current growth processes. To solve this issue, the N nutrition index (NNI) should be calculated at specific intervals during the vegetative growth period of crops [84]. As defined by Jeuffory and Bouchard [85] and Lemaire and Gastal [86], the NNI provides a basic tool for analyzing the actual plant N status in crops and can be used to quantify the levels of both the N deficiency and excess consumption of a given crop.

It seems that the N storage pools have an important function in improving N uptake efficiency when supplies are abundant (usually pre-anthesis), and buffer dwindling root uptake (usually post-anthesis). Two excellent reviews on this topic were published by Millard [87] and Barraclough et al. [81], according to which this extra nitrogen accumulation is achieved by producing infertile tillers and by storing N as nitrate, amino acids, amides and soluble proteins in various organs, tissues and organelles of fertile shoots [81]. In contrast, when N is deficient, the development of tillers is inhibited.

Ultimately, the N found in wheat grains generally comes from two sources: remobilization (N remobilization/NREM; originates from vegetative tissues) and assimilation (PANU; post-anthesis N uptake).

To date, several studies have been conducted following N-remobilization during the grain filling stage in wheat [29,88,89,90,91,92,93] mainly considering the remobilization from straw to grain. The results reported to date found that the amount of grain N that originated from the remobilization of pre-anthesis N is somewhere between 71.2% and 80% [56,94]. Moreover, Bogard et al. [48] concluded that both PANU and NREM contribute to NUE, but they found strongly negatively correlations among them. The same research found that just PANU significantly correlated with GPD, grain protein deviation [48].

In a research aiming to quantify the genetic variation in the uptake, Barraclough et al. [81] reported that in the partitioning and remobilization of N from vegetative organs to grain in a selection of 12 wheat varieties, there was a significant genetic variation in the N contents of different plant parts and its remobilization to grain, but there were no correlations, at a given N-rate, with the grain yield and quality.

### 3.2. NUE in Relation to G × E × M

The interactions between G × E × M should always be considered when evaluating NUE in a specific crop, including wheat, since the interaction of these factors constitutes the key point for designing sustainable and secure wheat crop yields.

The main purpose of the wheat breeding programs (G) developed to date, was mainly addressed to solve the problems related to the yield potential, improved quality and resistance to stress, both biotic and abiotic, by introducing new, mostly higher yielding varieties to compete and succeed in a commercial environment [95]. Nonetheless, genetics had a significant role to play in the development of more N-efficient plants, although the aim is not yet completed. The breeding programs developed to date, in this regard, led to a number of studies aiming not only to improve wheat crop yields, but also their NUE. The major purposes of the up-to-date studies were mainly focused on the partitioning of N between tissues at anthesis and the kinetics of senescence, which influence both the yield potential and nitrogen remobilization and partitioning [96]. The field experiments conducted to date on this topic, revealed that genetic variability for N uptake and NUE exists in small grains [97,98]. According to Cormier et al. [99], cited by Guttieri et al. [24], the Elite European winter wheat germplasm has demonstrated a trend toward improved NUE and the nitrogen harvest index (NHI) over the last 25 years. Other researchers [100,101] showed that semi-dwarf stature and flowering time significantly affect NUE traits for the Elite European winter wheat germoplasm. Barraclough et al. [81] developed research aiming to quantify the genetic variation in the uptake, partitioning and remobilization of N in individual plant organs at extreme rates of N supply (0 and 100 kg N ha^−1^) for twenty elite varieties of wheat. The research showed that grain yield and grain % N was well correlated with the plant N parameters (e.g., post-anthesis N-uptake, stem-N and vegetative-N at anthesis and vegetative-N remobilized), when compared to the two N-rates, but there was no correlation to the individual N-rates. Another research, conducted by Cui et al. [102], reported that the N-efficient cultivar on a 3-year on-farm study considering six N levels and two soft wheat cultivars (an N-efficient cultivar and a major commercial cultivar) showed a higher NUE compared to the major commercial cultivar, and a high growth and N uptake during post-anthesis, which demands a higher availability of N at later growth stages to achieve the maximum grain yield for this wheat cultivar [102]. A more complex study was conducted by Guo et al. [103], who followed the differences in the grain yield, NUE, root growth, root morphology, root distribution and nitrate transporter gene (TaNRT) expression. The authors of the study developed a 2-year microplot and sand culture experiment using 3 wheat cultivars (N-efficient, intermediate and N-inefficient), and their results showed that the N-efficient wheat cultivar had higher pre-anthesis root growth and N uptake ability, promoting leaf N assimilation ability and improving the wheat yield and NUE [103]. Considering the expression of the nitrate transporter gene, the authors of this study reported that TaNRT expression level at pre-anthesis was higher in the N-efficient cultivar than in the intermediate cultivar, whereas no significant genotypic difference was observed after anthesis [103], being associated with an increased grain yield and NUE.

Considering the influence of the environmental factors (E), the wheat grain yield response to fertilizer N is positively influenced by favorable light, temperature and water supply. Thus, the differences in these factors largely correlate with the grain yield and response to fertilizer N at different times of the year and from one year to another. The efficiency of a wheat genotype in relation to fertilizer N is also influenced by site location and characteristics, as different sites involve different soil types, temperature regimes and water supply. The available water capacity of the soil showed to have an important role in wheat grain yield efficiency and, thus, a positive relationship is found between the response to high rates of N application and the soil moisture status. In general, the amount of plant-available water reflects the water holding capacity of the soil in addition to the rainfall during the growing season. The potential effect of soil type in relation to the weather conditions also affects the wheat grain yield. Soils express the normal field capacity, generally at the beginning of the growing season, but the amount of plant-available water held at field capacity greatly differs between different soil types, for example, between sandy soils versus clay soils.

Fertilizer N is influenced by soil moisture as both the roots and the nutrients are generally found in high concentrations in the top layer of the soil, and thus the movement of nitrate and ammonium ions in this area by mass flow and diffusion requires adequate moisture. Therefore, in dry soil conditions occurring in the topsoil layer, wheat growth could be affected thanks to the restricted movement of N ions. The interactions between the water and N availability and the use efficiency should therefore be considered when evaluating NUE. Campbell et al. [104] conducted a 9-year experimental trial using zero-tillage management, on snow management by cereal trap strips and fertilizer N management, considering the rates, placement and timing of the application. The results showed that the efficiency of available N-use varied from 4 to 41 kg of grain kg^−1^ N, being directly correlated to the estimated water use and more generally to the available soil N, but inversely to the rate of fertilizer N. The authors of the study concluded that the fertilizer N placement (seed placed, deep band and broadcast) influenced the grain yields more than the timing, but in 5 out of 9 years neither factor was significant [104].

The environmental factors are almost unbreakable by the breeding programs since, thanks to them, we nowadays benefit from genotypes efficiently adapted to different soil–climate conditions in terms of yield and with a good or moderate NUE. Nonetheless, researchers need to be developed further considering also the current concerns and patterns specific to climate change, since N fertilization impacts and is impacted by this global issue. Considering that heat and drought are the major factors limiting wheat yield, further researches should firstly address the optimization of root-related traits.

To date, a significant number of field trials have addressed the issue of identifying the proper N management (M) that provides accurate fertilizer recommendations to improve NUE, i.e., an increase in the overall yield and reduction in the risk of environmental pollution. The major targets of these studies focused on the N timing and rate and the efficiency of the N uptake and recovery of wheat crops. Generally, the results achieved to date highlighted that on higher fertilizer rates more N is left in the soil (generally on N rates over 250 kg^−1^ N ha^−1^) and suggested some specific ranges of N supply that could serve as potential improvements. Nevertheless, this type of response should be validated for different environments and under different biotic and abiotic stress conditions, such that we can finally identify the proper N management that can deliver both a higher N efficiency and yields. Moreover, as we will further detail, there is a lack of knowledge regarding finding novel management practices and changes in the cropping systems, which could enhance higher NUE for wheat crops.

Thorup-Kristensen and Sørensen [100] developed a study that followed the dynamics of N in wheat crops and concluded that the N amounts left in the soil in the high N input plots reflected that the crop demand became saturated as the N fertilization increased. A similar study developed by Rasmussen et al. [61] revealed that in N inputs between 150 and 250 kg^−1^ N ha^−1^, more N was left in the soil, but at the same time more N was taken up, suggesting that within this range of N supply, there is the potential for future improvements, due to the improved root growth, improved root N uptake kinetics or improved shoot sink strength for N.

Wuest and Cassman [101] conducted a 2-yr field study aiming to compare the recovery of N fertilizer applied at planting (120, 180, or 240 kg N ha^−1^) with that applied at anthesis (0, 30, or 60 kg N ha^−1^), and evaluated the effects on the soil N uptake. Their results showed that the recovery of N applied at planting ranged from 30 to 55%, while that of N applied at anthesis ranged from 55 to 80%. The observations made on unfertilized N plots, revealed that the contribution of soil N to total plant N was not affected by the N rate or the timing of application [101].

Sowers et al. [105] evaluated the spring N applications conducted over four years on-site as an alternative to the application of N for improving NUE, exclusively during fall. Thus, the study compared five-to-six N rates (between 0 and 140 kg ha^−1^) for the fall N applications with the fall–spring split applications (between 84 to 140 kg total N ha^−1^), and a 15N experiment was conducted at two locations during the second year to quantify the N uptake from fertilizer and soil N [105]. The authors reported lower NUE values, with 26 to 44% in the N treatments with 140 kg ha^−1^ applied in fall, compared to the unfertilized control plot. On the other hand, their results showed for the plots on which N was applied with point injection or topdressing in spring NUE was maintained or increased, compared with the equivalent of all N rates (84 and 112 kg N ha^−1^) applied in fall. The authors of the study concluded that N applications in spring, with point injection or topdressing, can improve the recovery of N fertilizer and N use efficiency over preplant applications in dry land winter wheat [105]. Another field study conducted by Sowers et al. [106], concluded that under high residual soil N levels, reduced N rates and split N applications between fall and spring can maintain high yields and reduce grain protein.

Woolfolk et al. [107] conducted a 3-year study at 2 locations in Oklahoma, to evaluate the effects of late-season foliar N applications on the grain yield, total grain N, straw yield and total straw N. The foliar applications of N (in the form of urea ammonium nitrate, at the rates of 0, 11, 22, 34 and 45 kg N ha^−1^) were made at pre- and post-flowering, along with ammonium sulfate at a single rate of 22 kg N ha^−1^. Their results showed that late-season foliar N applications, before or immediately following flowering, may significantly enhance the grain N content and, thus, percent protein in winter wheat [107]. Similarly, Bly and Woodard [108] also highlighted through research that the grain protein and yield from plots without foliar N were inversely correlated. 

Rasmussen et al. [61] conducted a study aiming to evaluate the influence of N fertilization and winter wheat cultivar on the root growth, the utilization of inorganic N from deep soil layers and NUE. Their results showed that the effect of N fertilization on root density was mostly seen in the soil layers below 0.5 m. At the same time, the treatments seemed to have no effect on the root depth. The authors of the study concluded that one cultivar showed higher NUE compared to the other cultivars, highlighting that, under the studied experimental conditions, a spring application of up to 150 kg N ha^−1^ did not increase the amount of nitrate left in the soil at harvest. Further increases of N fertilization from 250 to 350 kg N ha^−1^ resulted in 90% of the extra N left in the soil.

## 4. Future Prospective for Improving NUE in Wheat Crops

With the current concerns related to the burgeoning population of the world and the proved-negative influence of the current wheat production patterns on the health of the ecosystem, scientists must develop more efficient N management strategies and more N-efficient wheat plants considering the specificities of the environment, i.e., considering the G × E × M interactions and complexities [24,28]. Therefore, plant breeding programs should focus on modifying the N uptake, utilization and remobilization of plant-available N [98]. Considering that wheat roots are generally more underdeveloped, compared to other cereals, and thus the solubilization of nutrients is lower (as we observed in the previous sections), these targets can be achieved by producing crops with denser and deeper root systems, which could enhance the rapid absorption of soil mineral N, and a greater competitive ability against microbes [6]. Theoretically, these research targets could constitute a viable solution in developing wheat genotypes that efficiently capture and convert available soil N into harvested GP, thus responding to the low N inputs and with greater NUE and grain yields. In practice, these objectives are not that easy to achieve, since screening for root variation in the field is extremely difficult, although methods are currently under development [109]. Moreover, the selection of high rooting biomasses can be beneficial in some environmental situations, but not others, depending on cost/benefit trade-offs [109]. The modification of nutrient uptake processes can also be achieved through the modifications of some enzymes, such as the alanine amino-transferase (AlaAT) enzyme [97]. Ultimately, N uptake improvement can be enhanced through the identification of specific root traits associated with N uptake and their underlying quantitative trait loci—QTL [98]. Nevertheless, there is a lack of knowledge in this topic since the QTL analyses conducted to date indicate a large number of small-effect QTLs for NUE traits [24,100]. Laperche et al. [98] identified six QTL associated with root architecture, and reported a large genetic variation in their double haploid population for N-specific uptake rates; therefore, although these researches can be considered as a starting point for future improvement, work still needs to be performed in this area to find a solution for this gap in the knowledge.

A sustainable means of management for N in wheat crops should be both efficient and effective, to deliver sufficient yields for the growing population worldwide, providing qualitative products while enhancing environmental protection. Thus, a high final protein content is desirable since this component constitutes a major determinant of wheat end-use value and baking quality [48]. Considering the negative genetic relationship expressed by grain yield and grain protein content, this task is difficult to accomplish, but different strategies both in the field of management practices and breeding programs can contribute to the success of this mission. Therefore, N management strategies should focus not only on identifying the optimum N rates for the different environmental conditions. These kinds of approaches deliver only half of our targeted objectives, and a viable solution should primarily focus on finding novel management practices with an increased potential to deliver all the desired and urgently needed solutions, in close collaboration with breeding programs and environmental specificities. Some of them have already proven their effectiveness, such as the precision agriculture that constitutes a viable solution for delivering N nourishment to wheat crops at the rate place, timing and rates considering all the specificities of a certain agro-ecosystem and, most importantly, soil N supply.

Considering the climate change context that enhances the unpredicted biotic and abiotic stresses, with variations from one year to another and even along one crop cycle, the use of agricultural unmanned aerial vehicles (UAVs) can also efficiently assist farmers in adopting the required interventions at the right timing, place and rate.

**Figure 2:**
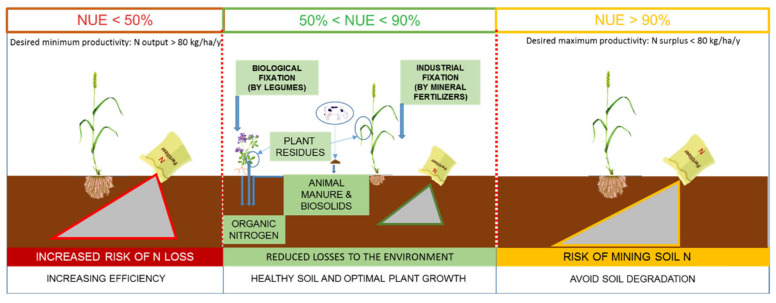
Ranges of desired NUE values (original).

Future programs should also focus on finding the changes in cropping systems that can be made, so that we can enhance the 3 Qs that are so urgently needed. The key point of all these practices should be promoting soil health because healthy soil can further enhance all of the 3Qs between the ranges of desired NUE values (50%–90%), as they were presented by the EU Nitrogen Expert Panel [110] (Figure 2).

**Figure 3:**
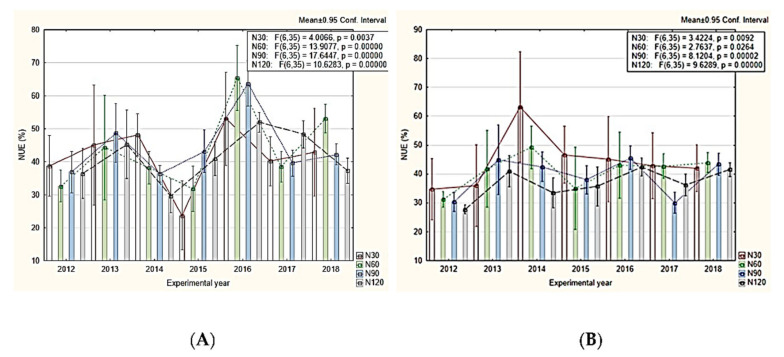
Main scenarios in NUE (reproduced after Vidican et al. [8] under the CC BY license). (**A**) wheat after maize and (**B**) wheat after soybean; N30, 60, 90 and 120 fertilization treatments with nitrogen (30, 60, 90 and 120 kg N ha^−1^ y^−1^). Means of six replicates: *p* values < 0.001 are highly significant from a statistical point of view (HS, confidence 99.9%).

This could be achieved through carbon farming practices that promote healthy soil and optimal plant growth through reduced/or without soil tillage, covering at least 30% of the land surface with vegetation, increasing agro-ecosystem biodiversity and balanced N application. This type of approach is also greatly promoted by the European Green Deal, with the final aim to reduce humankind’s environmental footprint. For the particular case of the wheat cropping system, one valuable practice could be the promotion of agroecosystem biodiversity through the introduction of crop rotations and cover crops. Legume–cereal intercropping systems can increase NUE and yield thanks to the ability of legumes to fix N from the atmosphere by biological N fixation [111]. Vidican et al. [8] showed in a 7-year study following the variations in NUE in a wheat–maize–soybean rotation crop, that an increase of 9.44% in the N uptake was recorded in wheat after soybean, compared to wheat after maize (Figure 3). Therefore, this kind of approach could constitute a viable solution for achieving the 3 Qs translated through an optimum NUE between 50% and 90%.

Other management strategies to improve the NUE in soil–plant–environment systems could be the application of plant–growth promoting rhizobacteria (PGPR) in soil [112] and the seed bio-priming process, using micro-organisms, such as the *Bacillus*, *Pseudomonas*, *Rhizobium* and *Trichoderma* species [112]. The use of PGPR could enhance the 3 Qs through promoting a higher efficiency in plant growth and tolerance to biotic and abiotic stresses, N use and N uptake. Ultimately, N management should also focus on finding the optimum splitting rates of fertilizer throughout the plant’s life cycle, so that the N applied could be used effectively in the key points of wheat development. To date, some researches are addressed to this type of approach, but there is limited information that correlates all these management strategies with breeding programs and environmental specificities.

## 5. Conclusions

The major concern related to wheat cropping systems nowadays resume in finding the optimum balance between the 3 Qs that are urgently needed, i.e., achieving a high quantity for the growing population worldwide, delivering good quality products and finding the quintessence for natural environment health enhancement. Considering that N fertilization is an indispensable management practice for wheat crops, but at the same time a major contributor to the pollution of the natural environment, optimizing NUE is of outstanding importance. There are numerous NUE indices available in the literature, making our mission to calculate wheat crop NUE even more difficult. Why? This is due to the fact that selecting and applying a certain NUE index should always rely on the particularities of our cropping system (the hypothesis of our research). Thus, we cannot provide a general solution that is applicable to all wheat cropping systems, this being attributed to each researcher depending on the question addressed by each study. Generally, the most commonly used indices are the fertilizer-based (PFP, AI, RE) and plant-based (PE, IE, NHI) NUEs, which mainly address short-time scales, such as the growing season. The soil-based indices (Nuptake, NUEyield) can provide valuable information regarding the NUE of wheat, and their assessment is required especially when the evaluation addresses a crop rotation. Finally, for long-term studies, we do recommend considering a calculation of the system-based indices, which could have an outstanding importance in evaluating the soil N over a period of time. With the current concerns related to the climate change issue, this mission is even more difficult because unpredicted biotic and abiotic stress are most likely to appear. Therefore, any viable solution stands in finding the ability to combine genetic, agronomic and environmental variables to the cropping system. To date, several improvement were made, so that we know for certain that: (i) plant breeding programs should focus on delivering more N-efficient cultivars (by modifying the N uptake, utilization and remobilization of plant-available N); (ii) studies should address the specificities of a certain environment so that we can further use these N-efficient plants with success on a worldwide scale, leaving no one behind and considering the climate change patterns; (iii) the management practices should focus on finding the optimum fertilization recipe for different environmental conditions (right place, timing, type of fertilizer and splitting doses among wheat life cycle in the key developmental stages), but also in identifying the changes that can be made in the cropping systems, enhancing healthier soils and thus a higher NUE (i.e., crop rotation and cover crops).

## Figures and Tables

**Table 1:** NUE indices and their calculation.

Approach	NUE Index	Calculation	Definition
**Fertilizer-based**	Agronomic efficiency [kg yield increase kg nutrient applied^−1^]	AE = (Y − Y_0_)/F	The productivity recorded as a result of N input [22,23]
	Partial factor productivity [kg harvested product kg nutrient applied^−1^]	PFP = Y/F	The productivity of the cropping system in relation to N inputs [22]
	Partial nutrient balance [kg harvested yield kg N applied^−1^]	PNB = U/F	The ratio between the amount of nutrients taken out of the system and the N applied [22]
	Apparent recovery efficiency [%]	RE = (U − U_0_)/F	The amount of nutrients applied assimilated by the plant [22,23]
**Plant-based**	Physiological efficiency [kg yield increase/kg increase in N uptake from the fertilizer]	PE = (Y − Y_0_)/(U − U_0_)	The ability of the plant to transform nutrients acquired from the source applied to economic yield [22]
	N Utilization efficiency [kg yield increase/kg increase in N uptake from the fertilizer]	NutE = Y/U	Similar to PE, but does not account for background N [21]
	Internal utilization efficiency [kg yield kg nutrient uptake^−1^]	IE = Y_N_/U	The fraction of plant tissue N that is contained in the yield component [21]
	N Harvest index [%]	NHI = (Y/U) × 100	The same as IE, but expressed as a percentage [21]
**Soil-based**	N Uptake efficiency [%]	NUpE = (U/F + S) × 100	The percentage of available soil N that is utilized by the plant; also conceptualized as the apparent recovery efficiency of the N supply [21]
	NUE_yield_	NUE_yield_ = NUpE × NUtE	The contribution of N supplied from the soil that is allocated to the yield N [21]
**Ecology-based**	Nitrogen productivity	NP = Relative growth rate/U	The ratio of the relative growth rate to the concentration of N in plant tissues [21]
	NUE_ecology_	NUE_ecology_ = NP × MRT	The product of N productivity and the mean residency time of plant N [21]
**System-based**	N Balance index of a system	Snbi = F − U − S	The accumulation or reduction in soil N over a set time
	NUE of a system (sNUE)	sNUE = Y_N_/Y_N_ + Nloss	The fraction of system N outputs that is captured as the N yield rather than lost to the environment [21]

Abbreviations: Y—crop yield with applied nutrients (kg/ha); Y_0_—crop yield (kg/ha) in a control plot, unfertilized with N; Y_N_—N in the yield component; U—total plant nutrient uptake in aboveground biomass at maturity (kg/ha) in a fertilized plot; U_0_—total nutrient uptake in aboveground biomass at maturity (kg/ha) in an unfertilized plot; F—fertilizer N; S—soil N; MRT—the mean residency time and NP—the product of N productivity.

## Supplementary Materials

The following are available online at https://www.mdpi.com/article/10.3390/plants11020217/s1, Table S1. List of abbreviations.
