# Supplementary material for: Current Status and Future Prospective for Nitrogen Use Efficiency in Wheat (Triticum aestivum L.)
Source: Plants (Basel). 2022 Jan 14;11(2):217. doi: 10.3390/plants11020217 (PMC8777959; doi:10.3390/plants11020217)
Supplement: Supplementary file 1 [file plants-11-00217-s001.zip › plants-1518980-supplementary.pdf]

Table S1: List of abbreviations

The following table describes the significance of abbreviations used throughout the manuscript, along with the page on which each one is defined or used for the first time. The most commonly used abbreviations for unit measure or chemical elements were not listed, but they are defined in the manuscript.

No. crt.	Abbreviation	Definition	Page
1.	GHG	Greenhouse gases emissions	1
2.	NUE	Nitrogen use efficiency	1
3.	The 3 Qs (original abbreviation)	Original abbreviation defined as High Quantity, good Quality and the Quintessence of natural environment health	1
4.	G	Genetics	1
5.	E	Environment	1
6.	M	Management	1
7.	EU	European Union	1
8.	EEA	European Environment Agency	1
9.	LULUC	Land use and land use change	1
10.	CAP	Common Agricultural Policy	2
11.	FAO	Food and Agriculture Organization	2
12.	MMT	Million metric ton	2
13.	AE	Agronomic efficiency	3
14.	PFP	Partial factor productivity	3
15.	PNB	Partial nutrient balance	3
16.	RE	Apparent recovery efficiency	3
17.	PE	Physiological efficiency	3
18.	NUtE	Nitrogen utilization efficiency	3
19.	Y	Crop yield	3

Continuation List of abbreviation

No. crt.	Abbreviation	Definition	Page
20.	Y0	Crop yield in a control plot, unfertilized	3
21.	U	Total plant nutrient uptake in aboveground biomass at maturity (kg/ha) in a fertilized plot	3
22.	U0	Total nutrient uptake in aboveground biomass at maturity (kg/ha) in an unfertilized plot	3
23.	F	Fertilizer N rate	3
24.	MRT	The mean residency time	3
25.	NP	The product of N productivity	3
26.	IE	Internal utilization efficiency	4
27.	NHI	Nitrogen harvest index	4
28.	NUpE	Nitrogen uptake efficiency	4
29.	NUE_{yield}	Nitrogen use efficiency in yield	4
30.	NP	Nitrogen productivity	4
31.	$NUE_{ecology}$	The product of N productivity and the mean residency time of plant N	4
32.	sNBI	N balance index of a system	4
33.	sNUE	Nitrogen use efficiency of a system	4
34.	C	Carbon	6
35.	NPF	Nitrate transporter 1/Peptide family	9
36.	NRT2	Nitrate transporter 2 family	9
37.	AMT	Ammonium transporters in plants	9
38.	ATP	Adenosine triphosphate	9
39.	NREM	N remobilization	10
40.	PANU	Post-anthesis N uptake	10
41.	GPD	Grain protein deviation	10
42.	TaNRT	Nitrate transporter gene	11
43.	AlaAT	The alanine amino-transferase	13
44.	QTL	Quantitative trait loci	13
45.	UAV	Unmanned aerial vehicles/Drones	14
46.	PGPR	Plant–growth promoting rhizobacteria	15
